# Acute hemodynamic and renal effects of glucagon-like peptide 1 analog and dipeptidyl peptidase-4 inhibitor in rats

**DOI:** 10.1186/s12933-015-0194-3

**Published:** 2015-03-07

**Authors:** Xiaoyan Zhou, Chin-hu Huang, Julie Lao, Alessandro Pocai, Gail Forrest, Olga Price, Sophie Roy, David E Kelley, Kathleen A Sullivan, Michael J Forrest

**Affiliations:** Department of Cardiometabolic Diseases, Merck Research Laboratories, 2000 Galloping Hill Road, Kenilworth, NJ 07033 USA; Janssen Research and Development, Cardiovascular and Metabolic Disease, 1516 Welsh and McKean Roads, Spring House, PA 19477 USA; In Vivo Pharmacology, Merck Research Laboratories, 2000 Galloping Hill Road, Kenilworth, NJ 07033 USA

**Keywords:** Glucagon-like peptide 1, Dipeptidyl peptidase-4, Hemodynamics, Renal function

## Abstract

**Background:**

Glucagon-like peptide 1 (GLP-1) analogs and dipeptidyl peptidase-4 (DPP4) inhibitors are a newer class of antidiabetics named as incretin-based therapy. In addition to the homeostatic control of glucose, the incretin-based therapy has shown beneficial effects on the cardiovascular system in preclinical and clinical studies. However, there is limited information on their renal effects. To this end, we assessed the acute hemodynamic and renal effects of a GLP-1 analog, Liraglutide, and a DPP4 inhibitor, MK-0626.

**Methods:**

Experiments were performed in anesthetized male Sprague–Dawley rats. Three ascending doses of Liraglutide (3, 9, and 27 nmol/kg/h) or MK-0626 (1 mg/kg) with or without GLP-1 peptide (2.4, 4.8, or 9.6 pmol/kg/min) were administered. Blood pressure (BP) and heart rate (HR) were recorded from an indwelling catheter. Glomerular filtration rate (GFR) and renal blood flow (RBF) were assessed by inulin and para-aminohippurate clearance, respectively. Renal excretory function was assessed in metabolic studies.

**Results:**

Both Liraglutide and MK-0626 plus GLP-1 evoked significant diuretic and natriuretic responses and increased GFR. MK-0626 alone increased RBF. Liraglutide at 27 nmol//kg/h and MK-0626 plus GLP-1 at 9.6 pmol/kg/min also increased HR, whereas BP was not affected.

**Conclusion:**

The results of the present study demonstrated that a GLP-1 analog and a DPP4 inhibitor may have beneficial effects on renal sodium and water handling. Additionally, the DPP4 inhibitor, MK-0626, favorably affects renal hemodynamics by increasing RBF. However, exceedingly high levels of GLP-1 receptor agonists may adversely affect the cardiovascular system in acute setting, as demonstrated by an acute increase in HR.

## Introduction

Incretins, including glucagon-like peptide-1 (GLP-1) and gastric inhibitory peptide, also known as glucose-dependent insulinotropic polypeptide (GIP) are a class of gastrointestinal hormones released from the small intestine in response to the presence of food. Incretins play an important role in the control of blood glucose through multiple mechanisms, predominantly via a glucose-dependent insulinotropic effect and via suppression of glucagon release [1]. The ability to exploit the natural biology of the incretins affords the opportunity to develop novel therapeutic options for the treatment of type II diabetes. The two primary classes of incretin-based therapies include (1) GLP-1 analogs (e.g., Exenatide and Liraglutide) that serve to mimic endogenous GLP-1 and (2) dipeptidyl peptidase-4 (DPP4) inhibitors (e.g. Sitagliptin, Saxagliptin, Linagliptin, and Vildagliptin) that inhibit the enzymatic degradation of GLP-1. In addition to their effects on the control of glucose, incretin-based therapies have demonstrated beneficial effects on the cardiovascular system and metabolism in preclinical and clinical studies [2-18]. For example, chronic administration of recombinant GLP-1 or the Exenatide analog AC3174 attenuated hypertension development in Dahl salt-sensitive (Dahl S) rats [2,3]. Sitagliptin attenuated blood pressure (BP) elevation in young prehypertensive spontaneously hypertensive rats (SHRs) [4]. Sitagliptin also decreased systolic BP, increased renal blood flow (RBF) [5], and improved endothelial function through reducing oxidative stress in adult SHRs [6]. DPP4 inhibitor, MK-0626, prevented diastolic dysfunction and reduced myocardial fibrosis [7] and improved neovascularization in mice [8]. Exenatide improved vascular function in rats [9]. Type II diabetic patients treated with Liraglutide or Exenatide or Vildagliptin exhibited a reduction in BP [10-12]. Saxagliptin improved microvascular function [13] and Liraglutide induced weight loss [14] in type II diabetes. In addition, recombinant GLP-1 infusion improved cardiac function in patients with chronic heart failure or with acute myocardial infarction and severe systolic dysfunction [15-18].

There is emerging evidence suggesting that some of the beneficial effects of incretin-based therapy on the cardiovascular system may be attributed to improvements in renal hemodynamics and renal function [19-25]. The underlying mechanisms responsible for these effects remain to be fully elucidated. To this end, we assessed the acute hemodynamic and renal effects of a GLP-1 analog, Liraglutide, and a DPP4 inhibitor, MK-0626. Due to the low level of endogenous GLP-1 under conditions of fasting [26], the effects of DPP4 inhibition were evaluated in the presence or absence of an infusion of exogenous GLP-1.

## Materials and methods

### Animals

Male Sprague–Dawley (SD) rats (Taconic, Petersburgh, New York) were housed in a temperature- and humidity-controlled facility with a 12 hour light: 12-hour dark, light cycle. Standard rat chow (#7012, Harlan Teklad, Madison, Wisconsin) and tap water were provided *ad libitum*. All procedures utilizing experimental animals were conducted in accordance with the Guide for the Care and Use of Laboratory Animals, and experimental procedures were reviewed and approved by the Institutional Animal Care and Use Committee at Merck Research Laboratories, Rahway, New Jersey.

### Reagents

GLP-1 was purchased from CPC Scientific (San Jose, California). MK-0626 was synthesized by Merck & Co., Inc.

### Experimental procedures

After one week of acclimation, SD rats (10–12 weeks old) were randomly divided into study groups. Rats were anesthetized with thiobutabarbital sodium (Inactin, 110 mg/kg, IP; Sigma-Aldrich, St. Louis, Missouri). Core temperature was maintained between 36 and 37°C throughout the study using a heating pad (Fine Science Tools Inc., Foster City, California). Polyethylene PE-50 tubing was inserted into the left femoral artery to permit intermittent blood sampling, and was also connected to a pressure transducer (NL108T2, Harvard Apparatus, Holliston, Massachusetts) for the measurement of blood pressure (BP) and heart rate (HR). The signal from the pressure transducer was transmitted to a Ponemah data acquisition system (Data Science International, St. Paul, Minnesota). The left femoral vein was cannulated with PE-50 tubing for solution administration and compound delivery, respectively. The bladder was catheterized with PE-100 tubing to allow timed collections of urine. Upon completion of the surgical procedures, sterile isotonic saline containing 1% albumin, 7.5% polyfructosan inulin (Fressenius Kabi Austria GmbH, Graz, Austria), and 1.5% para-aminohippuric acid (PAH) (Sigma-Aldrich, St. Louis, Missouri) was infused via the left femoral vein at a rate of 0.3 ml/100 g/h. In addition, 0.9% saline was infused via a Y shape adapter to the left femoral vein at a rate of 0.1 ml/100 g/h. Rats were allowed to stabilize for ~1 hour before initiation of the study. The experimental protocol was initiated with a 20-minute baseline urine collection, followed by administration of either vehicle or test compound at a rate of 0.1 ml/100 g/h for 60-minutes. At baseline and the end of the study, blood samples (500 - 600 μl) were collected for analysis of electrolytes (Na^+^, K^+^, and Cl^−^), hematocrit, active GLP-1 levels, and DPP4 activity. Urine volumes were measured and urine was analyzed for electrolytes (Na^+^, K^+^, and Cl^−^) and pH at multiple time points during the study. GFR and RBF were determined by inulin and PAH renal clearance at baseline and the end of the study. At the termination of each study, rats were euthanized by CO2 inhalation.

### Protocol design

#### Protocol 1: Liraglutide studies

**Figure 1:**
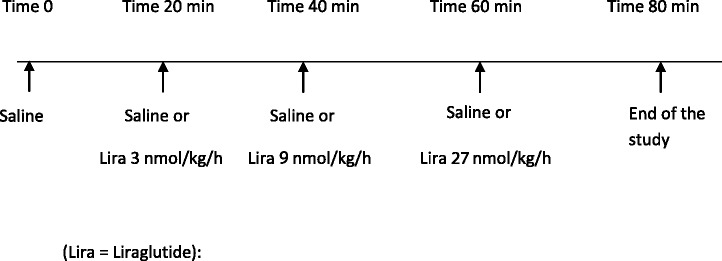
**Schematic of experimental protocol 1.**

The aim of the study was to evaluate the acute hemodynamic and renal effects of Liraglutide in anesthetized rats. One group of animals received four successive infusions of saline. A second group received an initial infusion of saline followed by three successive infusions of Liraglutide (Lira.) at ascending doses of 3, 9 and 27 nmol/kg/h (doses were selected based on a pilot study determining a minimal efficacious dose). All infusions were of 20 minutes duration for each dose. BP, HR, urinary Na^+^, K^+^, and Cl^−^ excretion were measured at baseline and at the end of each 20 minute infusion period. GFR and RBF were assessed at baseline and at the end of the last infusion period in each animal (Figure 1).

#### Protocol 2: MK-0626 with or without GLP-1 studies

**Figure 2:**
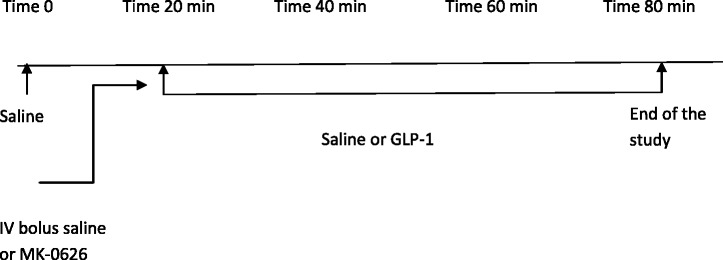
**Schematic of experimental protocol 2.**

The aim of the study was to evaluate the acute hemodynamic and renal effects of MK-0626 in the presence or absence of an infusion of exogenous GLP-1 in anesthetized rats. Animals were randomized into 8 experimental groups (n = 6 - 8) (Table 1): Group 1. Saline plus saline; Group 2. Saline plus GLP-1 2.4 pmol/kg/min; Group 3. Saline plus GLP-1 4.8 pmol/kg/min; Group 4. Saline plus GLP-1 9.6 pmol/kg/min; Group 5. MK-0626 1 mg/kg plus saline; Group 6. MK-0626 plus GLP-1 2.4 pmol/kg/min; Group 7. MK-0626 plus GLP-1 4.8 pmol/kg/min; and Group 8. MK-0626 plus GLP-1 9.6 pmol/kg/min. Saline or MK-0626 (1 mg/kg) was given by intravenous bolus injection immediately prior to saline or GLP-1 intravenous infusion. Parameters measured are the same as described in Protocol 1 (Figure 2).

**Table 1:** **Study groups in Figure** 2

**Groups**	**Treatment**
1	Saline (iv bolus) followed by saline
2	Saline (iv bolus) followed by GLP-1 @ 2.4 pmol/kg/min
3	Saline (iv bolus) followed by GLP-1 @ 4.8 pmol/kg/min
4	Saline (iv bolus) followed by GLP-1 @ 9.6 pmol/kg/min
5	MK-0626 (1 mg/kg) (iv bolus) followed by saline iv infusion
6	MK-0626 (1 mg/kg) (iv bolus) followed by GLP-1 @ 2.4 pmol/kg/min
7	MK-0626 (1 mg/kg) (iv bolus) followed by GLP-1 @ 4.8 pmol/kg/min
8	MK-0626 (1 mg/kg) (iv bolus) followed by GLP-1 @ 9.6 pmol/kg/min

iv. intravenous.

Doses selected for liraglutide and GLP-1 were based on the results from our pilot studies showing natriuresis/diuresis with plasma levels greater than the EC50 for activation of the GLP-1 receptor.

### Analytical techniques

Na^+^, K^+^, and Cl^−^ concentrations in plasma and urine were measured by a Roche Modular Chemistry System (Roche Diagnostics, Indianapolis, Indiana). Hematocrit was determined using a HESKA I-STAT Hematology Analyzer (HESKA, Fribourg, Switzerland). Urine pH was measured by a pH meter (Fisher Scientific, Pittsburgh, Pennsylvania). Inulin and PAH concentrations in plasma and urine were determined by colorimetric assays. The plasma concentration of active GLP-1 was determined using an Enzyme-Linked Immunosorbent Assay method (BioNebraska, Omaha, Nebraska). Plasma DPP4 activity was measured using a continuous fluorometric assay with the substrate Gly-Pro-AMC, which is cleaved by DPP4 to release the fluorescent AMC leaving group.

### Statistical analysis

All data are presented as mean ± standard error of the mean (SE). A paired Student’s *t*-test was used for comparisons of all treatment time points with baseline. A p value of < 0.05 is considered to be statistically significant.

## Results

### Hemodynamic and renal effects of Liraglutide

**Figure 3:**
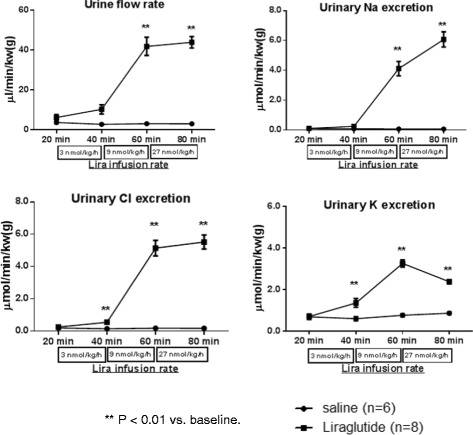
**Effects of Liraglutide on urine flow rate, urinary Na** ^**+**^ **, K** ^**+**^ **, and Cl** ^**−**^ **excretion.** Liraglutide was administered by three successive infusions of ascending doses of (3, 9 and 27 nmol/kg/h), 20 minutes for each dose. Data are mean ± SEM (n = 6 for saline group, n = 8 for Liraglutide group). ** P < 0.01 vs. baseline. Baseline refers to the 20 minutes period prior to administration of saline or Liraglutide.

Initial studies evaluated the acute hemodynamic and renal effects of Liraglutide. The intravenous infusion of Liraglutide (9 and 27 nmol/kg/h) significantly increased HR; BP remained unchanged at all doses of Liraglutide (Table 2). Liraglutide (9 and 27 nmol/kg/h) also evoked significant diuresis (6.7 and 7.0 fold), natriuresis (38.3 and 56.4 fold), chloruresis (20.3 and 22.9 fold), and kaliuresis (4.7 and 3.4 fold) (compared to pre-Liraglutide administration, all p < 0.01, Figure 3). Urine pH was also significantly increased from a baseline of 6.4 ± 0.1 to 7.1 ± 0.1 and 7.5 ± 0.1 at doses of 9 and 27 nmol/kg/h, respectively (p < 0.05). GFR and RBF were measured at baseline and at the conclusion of the last infusion of either saline or Liraglutide (27 nmol/kg/h). GFR was increased from 0.9 ± 0.09 at baseline to 1.4 ± 0.05 ml/min/kw(g) post-Liraglutide infusion (p < 0.05). RBF was not significantly different between baseline and post-Liraglutide infusion (Table 3). At the end of the study, the plasma K^+^ concentration was significantly decreased (3.3 ± 0.08 vs. 4.1 ± 0.15 mmol/l at baseline, p<0.05) and the hematocrit was significantly increased (43.9 ± 0.7 vs. 39.9 ± 0.4 at baseline, p<0.05) only in animals that received Liraglutide. Presumably these changes were a consequence of Liraglutide induced kaliuresis and volume contraction. At the end of the Liraglutide infusion, the plasma glucose level was slightly higher than at baseline (Table 3); we speculate this is attributable to the substantial diuresis/natriuresis induced by Liraglutide, which led to extracellular volume contraction and hemoconcentration.

**Table 2:** **Effects of Liraglutide (all three doses) on blood pressure and heart rate**

	**BP (mmHg)**	**HR (bpm)**
	**Baseline→treatment**	**Baseline→treatment**
Saline alone	121.8 ± 5.0 **→**116.0 ± 4.7	378.6 ± 9.5 **→**360.1 ± 6.3
Lira 3 nmol/kg/h	125.5 ± 1.5**→**124.9 ± 1.8	393.3 ± 7.8**→**397.9 ± 12.2
Lira 9 nmol/kg/h	125.5 ± 1.5**→** 127.3 ± 2.4	393.3 ± 7.8**→**415.0 ± 12.6*
Lira 27 nmol/kg/h	125.5 ± 1.5**→** 129.4 ± 1.8	393.3 ± 7.8**→**425.9 ± 14.3*

Lira, Liraglutide. *P < 0.05 post- vs. pre-treatment (baseline). In the saline alone group, BP and HR were not changed through the study; data shown in the table are the 1^st^ and last study period. Baseline refers to the 20 minutes period prior to administration of saline or Liraglutide.

**Table 3:** **Effects of Liraglutide (at the end of the study) on renal function, blood glucose and insulin**

	**Saline**		**Liraglutide**	
	**Baseline**	**Treatment**	**Baseline**	**Treatment**
GFR (ml/min/kw(g))	1.3 ± 0.16	1.5 ± 0.21	0.9 ± 0.09	1.4 ± 0.05*
RBF (ml/min/kw(g))	8.3 ± 0.49	10.5 ± 1.18	6.8 ± 0.99	8.4 ± 1.38
Glucose (mg/dl)	132.3 ± 3.33	140.4 ± 5.6	144.9 ± 4.2	168.9 ± 10.7*
Insulin (ng/ml)	2.2 ± 0.25	3.1 ± 0.62	3.0 ± 0.5	3.6 ± 0.7

*P < 0.05 post- vs. pre-treatment (baseline). Baseline refers to the 20 minutes period prior to administration of saline or Liraglutide.

### Hemodynamic and renal effects of MK-0626 and GLP-1

**Figure 4:**
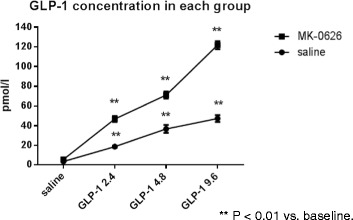
**Effects of exogenous GLP-1 infusion on plasma active GLP-1 level.** Data are mean ± SEM. ** P < 0.01 vs. baseline. Baseline refers to the 20 minutes period prior to IV administration of saline or MK-0626.

**Figure 5:**
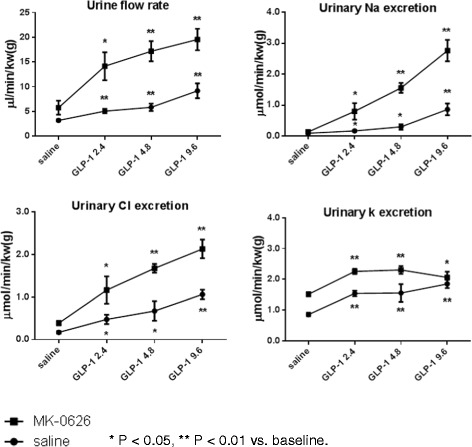
**Effects of MK-0626/GLP-1 on urine flow rate, urinary Na** ^**+**^ **, Cl** ^**−**^ **and K** ^**+**^ **excretion.** Data are mean ± SEM. *P < 0.05, **P < 0.01 vs. baseline. Baseline refers to the 20 minutes period prior to IV administration of saline or MK-0626.

**Figure 6:**
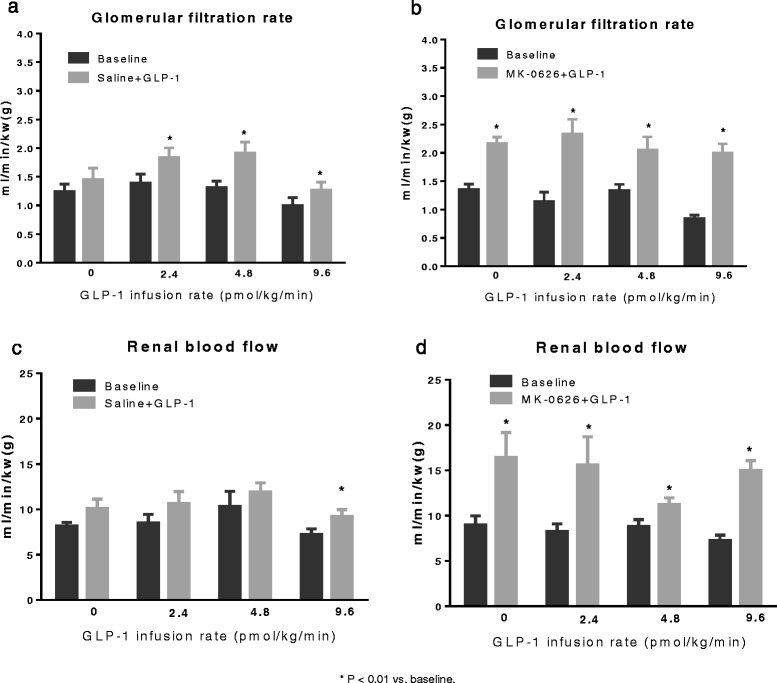
**Effect of MK-0626 and/or GLP-1 on GFR and RBF.** GFR, glomerular filtration rate. RBF, renal blood flow. **a** and **c**, effects of saline+GLP-1 on GFR and RBF, respectively. **b** and **d**, effects of MK-0626+GLP-1 on GFR and RBF, respectively. *p < 0.05 vs. baseline. Baseline refers to the 20 minutes period prior to IV administration of saline or MK-0626.

The effects of the DPP4 inhibitor, MK-0626, alone and in the presence of GLP-1, on hemodynamic and renal effects were evaluated. Baseline plasma GLP-1 levels are exceedingly low in fasted animals and hence are only marginally increased in the presence of DPP4 inhibition. Accordingly, exogenous GLP-1 was infused to achieve plasma GLP-1 levels similar to those observed following a meal. Plasma DPP4 activity was inhibited by greater than 90% in animals that received MK-0626 (1 mg/kg). The infusion of exogenous GLP-1 at doses of 2.4, 4.8 and 9.6 pmol/kg/min induced a step-wise increase in plasma active GLP-1 levels, which were further enhanced in animals previously administered MK-0626 (Figure 4). In animals that received MK-0626 plus the high dose of GLP-1 (9.6 pmol/kg/min), a significant increase in HR was observed (Table 4). BP and HR were not significantly affected by any of the other treatment paradigms of MK-0626, GLP-1 or combinations thereof. MK-0626 alone had no significant effect on either diuresis or natriuresis (Figure 5). GLP-1 alone induced a modest, albeit significant increase in diuresis, natriuresis, chloruresis and kaliuresis. However, MK-0626 plus increasing doses of GLP-1 administration evoked a dose dependent increase in diuresis, natriuresis, chloruresis and kaliuresis (Figure 5). Urine pH was also significantly increased by combinations of MK-0626 plus exogenous GLP-1 (Table 5). The effects of MK-0626 and GLP-1 on GFR and RBF are complex. GFR was increased in all animals that received MK-0626 but these effects were not further enhanced in the presence of increasing doses of GLP-1 (Figure 6b). In animals that received GLP-1 alone, there was a modest increase in GFR but the effects did not exhibit any dose-dependence (Figure 6a). A similar pattern of changes and absence of dose-dependence was observed for the effects of MK-0626 plus GLP-1 on RBF (Figure 6c,d). However, the magnitude of change was more variable with MK-0626 plus GLP-1. Furthermore, a significant effect of GLP-1 alone on RBF was only observed at the highest dose of GLP-1 (9.6 pmol/kg/min). The plasma K^+^ concentration was lower and the hematocrit was not significantly changed with MK-0626 plus various doses of GLP-1 administration (Table 6). Importantly, no significant changes in plasma insulin or glucose levels were observed at the end of study in animals that received MK-0626 plus GLP-1 administration (Table 7).

**Table 4:** **Effects of MK-0626 plus GLP-1 on blood pressure and heart rate**

	**BP (mmHg)**	**HR (bpm)**
	**Baseline→treatment**	**Baseline→treatment**
MK-0626 + saline	123.5 ± 3.7 →121.9 ± 3.9	381.2 ± 10.0 → 380.9 ± 10.3
MK-0626 + GLP-1 2.4 pmol/kg/min	121.1 ± 4.8 →118.7 ± 3.7	365.1 ± 12.6 →385.1 ± 8.6
MK-0626 + GLP-1 4.8 pmol/kg/min	125.7 ± 3.7 →123.5 ± 1.6	401.0 ± 6.5 →406.7 ± 9.2
MK-0626 + GLP-1 9.6 pmol/kg/min	124.7 ± 3.0→122.0 ± 2.3	386.9 ± 8.7→406.1 ± 8.5*

*P < 0.05 vs. baseline. Baseline refers to the 20 minutes period prior to IV administration of saline or MK-0626.

**Table 5:** **Effects of MK-0626 plus GLP-1 on urine pH**

	**Baseline**	**Treatment**
MK-0626 + saline	6.07 ± 0.17	6.25 ± 0.20
MK-0626 + GLP-1 2.4 pmol/kg/min	6.09 ± 0.14	6.93 ± 0.14*
MK-0626 + GLP-1 4.8 pmol/kg/min	6.03 ± 0.11	6.92 ± 0.12**
MK-0626 + GLP-1 9.6 pmol/kg/min	6.19 ± 0.11	6.90 ± 0.07**

*P < 0.05, **p < 0.01 vs. baseline. Baseline refers to the 20 minutes period prior to IV administration of saline or MK-0626.

**Table 6:** **Effects of MK-0626 plus GLP-1 on plasma K** ^**+**^ **and hematocrit**

**Group**	**K** ^**+**^ **(mmol/l)**	**Hematocrit (%)**
	**Baseline**→**treatment**	**Baseline**→**treatment**
MK-0626 + Saline	4.4 ± 0.14→4.2 ± 0.10	40.0 ± 0.8→38.2 ± 0.6
MK-0626 + GLP-1 2.4 pmol/kg/min	4.5 ± 0.08→3.8 ± 0.09**	40.1 ± 0.7→39.7 ± 0.5
MK-0626 + GLP-1 4.8 pmol/kg/min	4.4 ± 0.09→3.6 ± 0.05**	39.6 ± 0.6→39.8 ± 0.5
MK-0626 + GLP-1 9.6 pmol/kg/min	4.4 ± 0.10→3.6 ± 0.08**	40.5 ± 0.5→41.1 ± 0.6

**p < 0.01 vs. baseline. Baseline refers to the 20 minutes period prior to IV administration of saline or MK-0626.

**Table 7:** **Effects of MK-0626 plus GLP-1 on glucose and insulin**

	**Glucose (mg/dl)**	**Insulin (ng/ml)**
	**Baseline→treatment**	**Baseline→treatment**
MK-0626 + Saline	134.9 ± 6.0 **→**135.8 ± 7.3	2.5 ± 0.2 **→**2.7 ± 0.1
MK-0626 + GLP-1 2.4 pmol/kg/min	120.6 ± 7.6 **→**142.7 ± 14.7	2.7 ± 0.5 **→**2.2 ± 0.2
MK-0626 + GLP-1 4.8 pmol/kg/min	136.5 ± 7.9 **→**136.1 ± 8.8	1.8 ± 0.2 **→**1.5 ± 0.2
MK-0626 + GLP-1 9.6 pmol/kg/min	122.6 ± 4.1**→**131.1 ± 4.5	2.2 ± 0.2 **→**2.6 ± 0.4

Baseline refers to the 20 minutes period prior to IV administration of saline or MK-0626.

## Discussion

Modulation of the incretin axis as a therapeutic option for the treatment of type II diabetes has achieved considerable success. Emerging evidence indicates that these approaches not only have beneficial effects on metabolic parameters but also have favorable effects on cardiovascular function [2-18]. The studies described herein have focused on the acute effects of GLP-1 on hemodynamics and renal function in anesthetized normotensive and normoglycemic rats. The studies compared a metabolically stable GLP-1 analog, Liraglutide, with a DPP4 inhibitor, MK-0626.

One of the principal findings of these studies was that elevated circulating levels of active GLP-1 or the GLP-1 analog, Liraglutide, evoked a concentration dependent increase in both diuresis and natriuresis. Circulating levels of GLP-1 in man in the fasted state are approximately 10 pmol/L; but increase rapidly upon food intake to approximately 50 pmol/L [26]. GLP-1 is rapidly inactivated within the circulation predominately by DPP4 and has a circulating half-life of only 1 to 2 minutes. DPP4 inhibitors raise circulating plasma levels of GLP-1 by preventing its proteolytic degradation [27-29]. In the studies reported herein, baseline plasma GLP-1 concentrations are very low (<10 pmol/L; Figure 4), consequently administration of the DPP4 inhibitor MK-0626 alone only marginally increased circulating GLP-1 levels to 5.5 pmol/L from 3.4 pmpol/L. Therefore, in order to increase circulating GLP-1 to levels observed in the post-prandial state, animals were infused with exogenous GLP-1 either alone or following administration of MK-0626. Under these conditions, plasma active GLP-1 levels increased in proportion to the GLP-1 infusion rate. Furthermore, in the presence of MK-0626 at a dose that inhibits DPP4 activity by >90%, plasma active GLP-1 levels were similar to or greater than those measured in the postprandial state (approximately 40 - 60 pmol/l in rats). Importantly, the increased plasma levels of GLP-1 exceeded the concentration of GLP-1 (10 pmol/L) required for half maximal activation of the rat GLP-1 receptor (EC50) in vitro (unpublished internal data). Our observation, that both diuresis and natriuresis positively correlated with plasma GLP-1 levels (Figures 4 and 5), is consistent with the renal effects being mediated by GLP-1 activation of the GLP-1 receptor. This contention is further supported by results of the studies with Liraglutide. In these studies increased diuresis and natriuresis were observed at Liraglutide doses of 9 and 27 pmol/Kg/h which achieved Liraglutide concentrations 18 and 50-fold above the EC50 value for activation of the GLP-1 receptor by Liraglutide.

The mechanism of the natriuresis and diuresis evoked by Liraglutide and MK-0626 plus GLP-1 is not fully understood but is likely attributable to effects on the renal tubules. The contribution to natriuresis and diuresis by an increase in GFR appears to be minimal as the GFR increase by either Liraglutide or MK-0626 plus GLP-1 is modest and is also not dose-dependent. We speculate that the renal tubular effects of Liraglutide or MK-0626 plus GLP-1 may be mediated, at least in part, by GLP-1 receptor activation and consequent inhibition of sodium hydrogen exchanger 3 (NHE_3_). There are several lines of evidence supporting our contention: 1. GLP-1 receptor mRNA and protein are expressed in the proximal tubular cells in rats and pigs [20,30,31]; while a recent study reported that the GLP-1 receptor is exclusively localized in the smooth muscle cells of arterioles and blood vessels lining the kidney in the primates and humans [32]. The discordant results for GLP-1 receptor localization could be due to differences in detection techniques or represent species-specific differences. 2. GLP-1 receptor agonism reduces NHE_3_ activity and inhibits sodium re-absorption in renal proximal tubule cells [31,32]. 3. Renal microperfusion studies showed that GLP-1 significantly decreases NHE_3_-mediated bicarbonate reabsorption in the intact renal proximal tubule [20]. 4. Intravenous infusion of GLP-1 in anesthetized rats increased urine flow and urinary excretion of sodium, potassium, bicarbonate [19,20] and also increased fractional excretion of lithium [19], an index of proximal tubular reabsorption [33]. Finally, our observation of an increase in urine pH in animals that received Liraglutide or MK-0626 plus GLP-1 is consistent with blockade of a sodium-hydrogen exchange mechanism.

In addition to renal tubular effects, Liraglutide, GLP-1, or MK-0626 plus GLP-1 promoted an increase in GFR. The mechanism or mechanisms for the increase in GFR are not clear. One potential contribution is through dilatation of renal afferent arterioles. The GLP-1 receptor is widely expressed in the vasculature [34,35]; GLP-1 causes vasorelaxation in the isolated rat aorta and femoral artery [36,37] and increases blood flow in the forearm or the brachial artery in humans [38,39]. Importantly, the GLP-1 receptor is expressed in the renal artery [5,40], and as shown by others and in the studies described here, GLP-1 and GLP-1 receptor agonists increase RBF in anesthetized rat studies [19,20]. The absence of a dose-dependent effect of either GLP-1 or Liraglutide on either GFR or RBF indicates that increased RBF is not the sole determinant of the increase in GFR. Glomerular filtration is regulated and maintained by numerous physiological processes. In particular, changes in tubuloglomerular feedback (TGF) could counteract other influences on GFR [41,42]. The increased delivery of sodium chloride to the macula densa as a result of inhibiting NHE_3_ in the proximal tubule by GLP-1 receptor activation should trigger enhancement of TGF and hence a reduction of GFR. Indeed, a proximal tubule diuretic such as benzolamide activates TGF and lowers GFR [43]. Moreover, natriuresis and diuresis may result in volume contraction and activation of the sympathetic nervous system and renin-angiotensin aldosterone system [44-46], both of which could lower GFR. Clearly, a more detailed assessment of the effects of GLP-1 receptor activation with respect to effects on renal vascular tone, TGF, neurohormonal activation and the interplay for these responses is warranted to fully understand the observed changes in GFR and RBF.

It is interesting to note that MK-0626 alone increased RBF even in the absence of GLP-1 administration. This raises the possibility of a GLP-1 receptor independent mechanism contributing to the RBF effect of MK-0626. Although GLP-1 is the primary DPP-4 substrate, DPP-4 also cleaves multiple peptides including GIP, stromal cell derived factor type 1 α (SDF1α), neuropeptide Y (NPY), peptide YY (PYY), B-type (brain) natriuretic peptide (BNP), glucagon like peptide-2, and other substrates [47]. Direct effects of these peptides on renal hemodynamics were not tested in the present study. Future studies are warranted.

Liraglutide at the highest dose administered (27 nmol/kg/h) and the combination of MK-0626 with a high dose of GLP-1 (9.6 pmol/kg/min) evoked significant increases in heart rate while not affecting blood pressure. These observations are consistent with persistent small increases in HR reported in clinical studies with Liraglutide [48]. Similarly, MK-0626 plus the utilized highest GLP-1 dose also increased HR. Our internal experiments demonstrated the EC50 of Liraglutide on rat GLP-1 receptor is 2.2 nmol/l (unpublished data). As mentioned previously administration of Liraglutide (27 nmol/kg/h) achieved a plasma concentration of 110.5 ± 18.8 nmol/l (50-fold greater than the EC50 for activation of the GLP-1 receptor by Liraglutide). Similarly administration of MK-0626 plus GLP-1(9.6 pmol/kg/min) achieved a plasma concentration of active GLP-1 of 121.8 ± 11.5 pmol/L (12-fold greater than the EC50 for activation of the GLP-1 receptor by GLP-1). Whether these levels of Liraglutide or GLP-1 are sufficient to directly activate GLP-1 receptors in the heart or potentially in the CNS are unclear. Alternatively, these high levels of Liraglutide or GLP-1 may be activating other GLP-1 related receptors to evoke an increase in heart rate.

To summarize, we have shown that the GLP-1 analog, Liraglutide, and a DPP4 inhibitor, MK-0626, produced favorable effects on sodium and water handling, which is likely achieved via GLP-1 receptor activation in the renal proximal tubules. Exceedingly high levels of GLP-1 receptor agonists which activate GLP-1 receptors may contribute to the acute HR increase. Additionally, we also have shown that MK-0626 acutely increased RBF, which appears to be mediated, at least in part, by a GLP-1 receptor independent mechanism. Our findings suggest that a GLP-1 analog or a DPP4 inhibitor may provide additional renal effects beyond glycemic control. Although convincing, there are a few limitations. First, the present studies were performed in acute settings (mostly 20-minute for each observation period). Second, normotensive and normoglycemic rats were utilized in the present studies. Third, study animals were anesthetized in order to monitor renal function by collecting urine from bladder precisely through the study. Therefore, the findings from the present studies may not necessarily apply to chronic setting or in diseased animal models. Further chronic studies particularly in diabetic models or in models of renal dysfunction are warranted.

## Abbreviations

BP: Blood pressure
BNP: B-type natriuretic peptide
Dahl S rats: Dahl salt-sensitive rats
DPP4: Dipeptidyl peptidase-4
GFR: Glomerular filtration rate
GIP: Glucose-dependent insulinotropic polypeptide
GLP-1: Glucagon-like peptide 1
HR: Heart rate
Lira: Liraglutide
NHE3: Sodium hydrogen exchanger
NPY: Neuropeptide Y
PAH: Para-aminohippuric acid
PYY: Peptide YY
RBF: Renal blood flow
SD rats: Sprague–Dawley rats
SDF1α: Stromal cell derived factor type 1α
SHRs: Spontaneously hypertensive rats
TGF: Tubuloglomerular feedback
